# Draft Genome Sequence of *Ochrobactrum pseudogrignonense* Strain CDB2, a Highly Efficient Arsenate-Resistant Soil Bacterium from Arsenic-Contaminated Cattle Dip Sites

**DOI:** 10.1128/genomeA.00173-13

**Published:** 2013-04-18

**Authors:** Yiren Yang, Xuefei Yu, Ren Zhang

**Affiliations:** School of Biological Sciences, University of Wollongong, Wollongong, NSW, Australia

## Abstract

We report the 4.97-Mb draft genome sequence of a highly efficient arsenate-resistant bacterium, *Ochrobactrum* sp. strain CDB2. It contains a novel arsenic resistance (*ars*) operon (*arsR*-*arsC1*-*ACR3*-*arsC2*-*arsH*-*mfs*) and two non-operon-associated *ars* genes, *arsC3* and *arsB*. The genome information will aid in the understanding of the arsenic resistance mechanism of this and other bacterial species.

## GENOME ANNOUNCEMENT

An *Ochrobactrum* strain (CDB2) isolated from arsenic-contaminated cattle tick dip sites in northeastern New South Wales, Australia, exhibited high resistance to arsenical compounds, especially arsenate (1). Obtaining the full genome sequence of this organism would help us to understand the mechanism of arsenic resistance of this bacterium and related species.

A pair-end library was constructed and pyrosequencing was performed using a Roche GS FLX+ sequencer at the John Curtin School of Medical Research, the Australian National University, Canberra. After assembling the raw data by using the GS *de novo* Assembler (Roche, version 2.6.3), we used the RAST (Rapid Annotations using Subsystems Technology) server (2) for draft genome annotation, subsystem classification, and G+C content calculation. The putative *ars* genes were further analyzed with BLASTp and HHpred (http://toolkit.tuebingen.mpg.de/hhpred). The NCBI Prokaryotic Genomes Automatic Annotation Pipeline (PGAAP) (http://www.ncbi.nlm.nih.gov/genomes/static/Pipeline.html) was used to produce GenBank format data.

The draft genome of *Ochrobactrum* sp. strain CDB2 comprises 4,971,228 bases assembled from a 20-fold coverage of raw data and has a G+C content of 53.6%. It contained 147 contigs, with the longest consisting of 350,235 bases while the median length of all contigs was 25,233 bases. We found 4,868 coding sequences (CDSs) and 54 tRNA/rRNA genes in the constructed genome.

Our previous identification at the genus level was based on an analysis of a partial 16S rRNA gene sequence (1,341 bases) and a fatty acid composition profile (1). With the full 16S sequence obtained, a new search showed a 100% match with a 1,387-bp 16S sequence from *Ochrobactrum pseudogrignonense* strain CCUG 30717^T^ (accession no. AM422371) (3). The nucleotide sequences of three other genes, *groEL, gyrB*, and *recA* (accession no. FM863822, FM863816, and AM422877, respectively), also matched perfectly, except for one base in *groEL*, between CDB2 and *Ochrobactrum pseudogrignone* CCUG 30717^T^. These identified CDB2 as a strain of *Ochrobactrum pseudogrignonense*.

Analysis revealed a novel *ars* operon (*arsR*-*arsC1*-*ACR3*-*arsC2*-*arsH*-*mfs*). Interestingly, two arsenate reductase genes coexist in it: the deduced protein ArsC1 was highly homologous to the ArsC of *Saccharomyces cerevisiae* (4), while ArsC2 more closely matched the *Escherichia coli* reductase (5). The last gene (*mfs*) is predicted to specify a protein belonging to the superfamily of major facilitator transporters while sharing only limited homology to known ArsB and ACR3 proteins. The function of this transmembrane transporter warrants investigation. We noticed that such an operon also exists in the *Ochrobactrum anthropi* strain ATCC 49188 genome (accession no. NC_009668.1, locus 521,109 to 525,495). A similar operon (lacking *mfs*) has been identified in *Ochrobactrum tritici* strain SCII24^T^ (6).

Two other putative *ars* genes, *arsC3* (which, like *arsC2*, also codes for a glutaredoxin-dependent arsenate reductase) and *arsB*, were also found in the genome. However, rather than being located in *ars* operons, each of them presents independently of other correlated *ars* genes. The existence of genes for three arsenate reductases and three transporters in the same genome may explain the remarkably high resistance of this bacterium to arsenate (1).

### Nucleotide sequence accession numbers. 

The draft annotated genome sequence has been deposited at DDBJ/EMBL/GenBank under the accession number AKVI00000000. The version described in this paper is the first version, AKVI01000000.

## References

[1] Chopra BK Bhat S Mikheenko IP Xu Z Yang Y Luo X Chen H van Zwieten L Lilley RM Zhang R 2007 The characteristics of rhizosphere microbes associated with plants in arsenic-contaminated soils from cattle dip sites. Sci. Total Environ. 378:331–342 17407787 10.1016/j.scitotenv.2007.02.036

[2] Aziz RK Bartels D Best AA DeJongh M Disz T Edwards RA Formsma K Gerdes S Glass EM Kubal M Meyer F Olsen GJ Olson R Osterman AL Overbeek RA McNeil LK Paarmann D Paczian T Parrello B Pusch GD Reich C Stevens R Vassieva O Vonstein V Wilke A Zagnitko O 2008 The RAST server: rapid annotations using subsystems technology. BMC Genomics 9:75 18261238 10.1186/1471-2164-9-75 PMC2265698

[3] Kämpfer P Scholz HC Huber B Falsen E Busse HJ 2007 *Ochrobactrum haematophilum* sp. nov. and *Ochrobactrum pseudogrignonense* sp. nov., isolated from human clinical specimens. Int. J. Syst. Evol. Microbiol. 57:2513–2518 17978211 10.1099/ijs.0.65066-0

[4] Chen CM Misra TK Silver S Rosen BP 1986 Nucleotide sequence of the structural genes for an anion pump. The plasmid-encoded arsenical resistance operon. J. Biol. Chem. 261:15030–15038 3021763

[5] Mukhopadhyay R Shi J Rosen BP 2000 Purification and characterization of Acr2p, the *Saccharomyces cerevisiae* arsenate reductase. J. Biol. Chem. 275:21149–21157 10801893 10.1074/jbc.M910401199

[6] Branco R Chung AP Morais PV 2008 Sequencing and expression of two arsenic resistance operons with different functions in the highly arsenic-resistant strain *Ochrobactrum tritici* SCII24T. BMC Microbiol. 8:95 18554386 10.1186/1471-2180-8-95 PMC2440759

